# Ecological evolution in a semi-arid lake: insights from subfossil diatoms and geochemical indicators in Hulun Lake

**DOI:** 10.3389/fmicb.2025.1550555

**Published:** 2025-03-31

**Authors:** Dunping Sun, Bin Xue, Shuchun Yao, Xiangzhen Kong, Can Zhang, Chen Du, Yufei Xue, Yiran Zhang

**Affiliations:** ^1^State Key Laboratory of Lake Science and Environment, Nanjing Institute of Geography and Limnology, Chinese Academy of Sciences, Nanjing, China; ^2^College of Resources and Environment, University of Chinese Academy of Sciences, Beijing, China; ^3^Key Laboratory of Lake and Watershed Science for Water Security, Nanjing Institute of Geography and Limnology, Chinese Academy of Sciences, Nanjing, China

**Keywords:** Hulun Lake, sedimentary, diatom, geochemical indicator, eco-environment evolution

## Abstract

Hulun Lake, one of the largest inland lakes in the grassland region of northern China, has undergone distinct ecological changes over the past century due to both natural climatic shifts and human activities. Despite its ecological significance, the long-term drivers behind these changes are still not fully understood, especially the interactions between climate and anthropogenic influences on lake dynamics. To fill this gap, we analyzed sediment core from Hulun Lake, examining subfossil diatom assemblages, geochemical indicators, and sediment characteristics to reconstruct environmental changes and uncover the mechanisms driving them. Our findings reveal a shift from predominantly planktonic to periphytic/benthic diatom communities, associated with changes in nutrient levels and hydrological conditions. Key indicators, such as total phosphorus (TP) and sand content, showed strong correlations with diatom community composition, indicating that nutrient influx and water level fluctuations play crucial roles in lake ecosystem dynamics. Before 1935 AD, Hulun Lake’s ecology was primarily driven by natural climatic variations, supporting eutrophic species in stable, nutrient-rich conditions. From 1935 to 1970 AD, nutrient levels rose gradually, with parts of human impact. However, since 1970 AD, as human activities decrease and the warming and drying trend of rising temperature, reduced precipitation has led to a significant drop in the lake water level and a shrinking water area, which of them have significantly influenced nutrient dynamics and diatom composition. This study underscores the combined effects of climate change and human activities in driving the ecological evolution of Hulun Lake, providing valuable insights for the future conservation and management of similar semi-arid lake ecosystems.

## 1 Introduction

Lakes in arid and semi-arid regions are crucial ecosystems that provide essential services, including water storage, biodiversity support, and socioeconomic benefits (Grant et al., 2021; Zhang et al., 2017). However, in northern China, climate change and human activities have led to significant shrinkage of many lakes, resulting in a decline in water levels, habitat loss, and threats to biodiversity (Pekel et al., 2016; Singh and Chudasama, 2021; Zhang C. et al., 2023; Zhang Z. et al., 2023). Understanding the driving factors behind these changes is vital for developing effective lake management strategies, with implications for policy-making aimed at mitigating ecological degradation and ensuring sustainable use of lake resources (Hu et al., 2017; Huang et al., 2017).

Previous studies have primarily relied on instrumental records and remote sensing to investigate lake shrinkage in China (Cao et al., 2021; Ho et al., 2019; Zhang et al., 2019). These studies highlight both climate variability and anthropogenic activities as key drivers of lake dynamics. However, such data often span limited time periods, introducing uncertainty into long-term analyses of whether climate change or human activities are the dominant factors (Jiang et al., 2017). For example, while remote sensing provides useful data on recent trends, it does not capture historical changes in lake ecosystems (Gaiser et al., 2009). To overcome this limitation, paleolimnological methods can be used to obtain the long-term patterns of ecological evolution (Bertini, 2006; Luniger and Schwark, 2002).

Paleolimnology, through the study of biological indicators preserved in lake sediments, offers valuable insights into long-term ecological changes. Among these indicators, diatoms are particularly effective due to their sensitivity to environmental variables such as water level and nutrient availability. Their siliceous cell walls, which differ in size, shape, and surface ornamentation, allow species-level differentiation, making them powerful tools for reconstructing past aquatic environments (Thacker et al., 2023). Consequently, shifts in diatom assemblages provide critical evidence for assessing the condition of freshwater ecosystems (Dilworth et al., 2023; Hamilton et al., 2021; Peng et al., 2022). On a broader scale, numerous studies have demonstrated the utility of diatoms in tracking environmental changes across diverse lake systems. For instance, research on Cuogeda Lake on the Tibetan Plateau identified significant ecological transformations over the past 4,000 years, primarily driven by climatic shifts and monsoonal variations (Zhang et al., 2024). Similarly, a study of a sinkhole lake in Florida linked diatom assemblage changes to both climate variability and human-induced land-use modifications (Quillen et al., 2013). In Lake Baikal, diatom records have documented profound community shifts in response to 20th-century warming, with endemic species being replaced by cosmopolitan taxa, highlighting the ecological consequences of rising temperatures (Roberts et al., 2019). Moving to more region-specific studies, Liao et al. (2020) examined diatom records from Basomtso in the southeastern Tibetan Plateau, suggesting that aquatic ecosystems in alpine lakes may face greater risks of regime shifts than terrestrial ecosystems under future climate scenarios. In northwest China, Lin et al. (2018) combined geochemical proxies with diatom analyses from Kanas Lake sediments to evaluate climate impacts on the lake ecosystem over the past century. These studies underscore the effectiveness of diatom-based reconstructions in understanding both long-term climatic influences and more recent anthropogenic pressures on freshwater systems.

Despite the proven utility of diatoms, few studies have applied them to the inland freshwater lake in the arid area. As one of the largest arid zone freshwater lakes in northern China, Hulun Lake, has undergone significant ecological changes. To bridge this gap, our study utilizes subfossil diatom records, geochemical indices, and grain size data to reconstruct Hulun Lake’s ecological evolution over the past century. We also compared the reconstruction results with paleoclimate records from neighboring regions in an attempt to elucidate the relationship between long-term ecological shifts in Hulun Lake and regional climate variations as well as anthropogenic activities. By combining these proxies, we aim to clarify the relative roles of climate change and human activities in driving long-term ecological dynamics in arid and semi-arid regions. This research will contribute to a better understanding of lake ecosystem responses to environmental stressors and inform future conservation efforts.

## 2 Materials and methods

### 2.1 Study area

**Figure 1:**
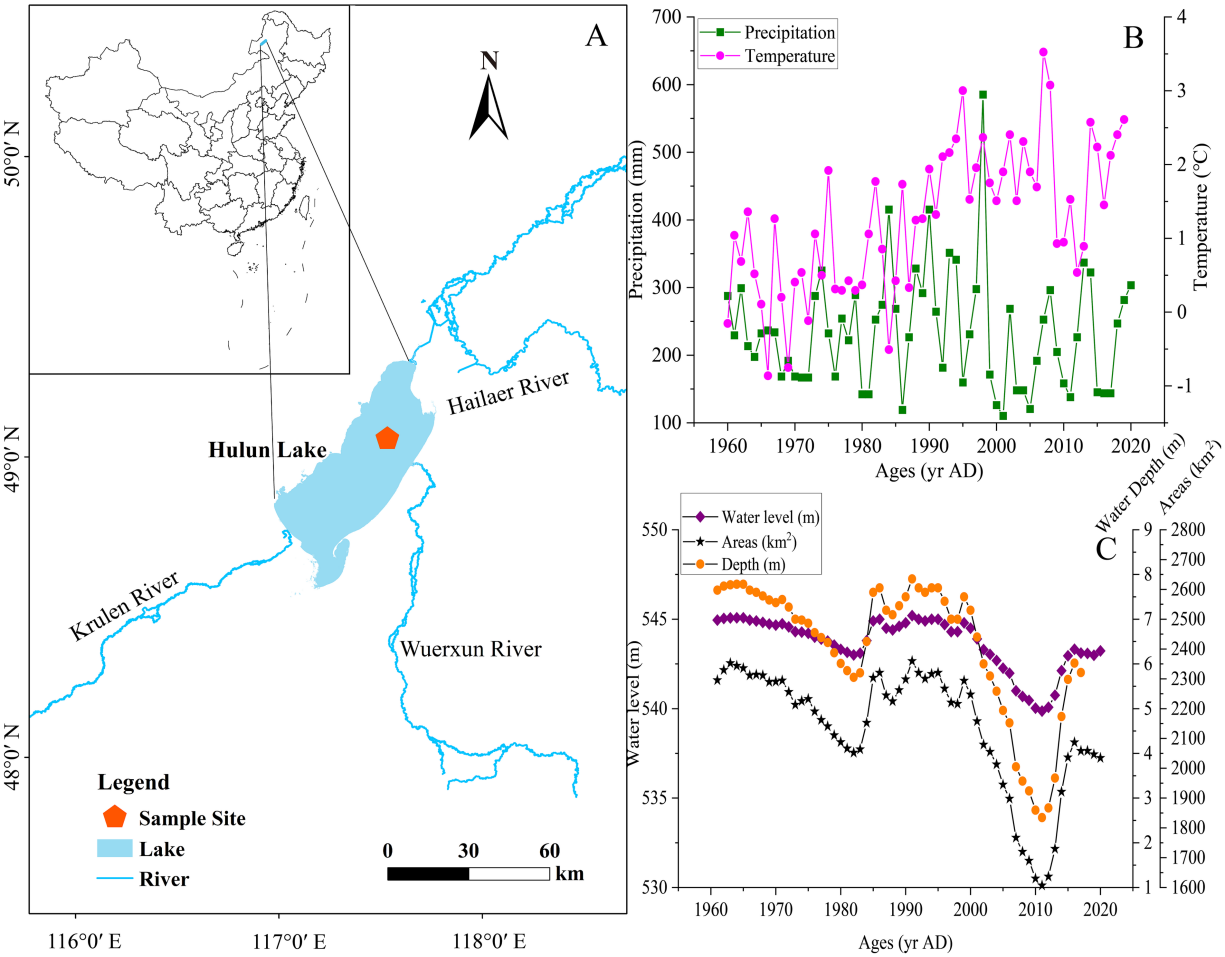
Map of the study area in northeast China, illustrating: **(A)** The location of Hulun Lake and the sediment core sampling site; **(B)** Meteorological data trends for Hulun Lake, including temperature and precipitation changes; **(C)** Hydrological data trends for Hulun Lake, showing water level, areas, water depth variations. Data sourced from the National Nature Reserve Administration of Hulun Lake.

Hulun Lake (48°30′40′′-49°20′40′′N, 117°00′10′′-117°41′40′′E), located in the western part of the Hulun Buir grassland in Inner Mongolia (Wu et al., 2015), is the largest lake in northern China (Zhang et al., 2018). It is fed by three major rivers: the Krulen River, the Wuerxun River, and the Hailaer River, entering from the southwest, southeast, and northeast, respectively. The lake has an irregular and oblique shape, with a length of 93 km and a width of 32 km, respectively (Figure 1A). The region belongs to a typical temperate continental climate, and 90% of the area covered by grassland (Xiao et al., 2009).

Since 1960 AD, the average annual temperature in the Hulun Lake Basin has been 1.3°C, and the ice-covered period lasts approximately 6 months. The mean annual precipitation is 235 mm, with large evaporation rates and low rainfall (Figure 1B). Precipitation is mainly concentrated from June to September, accounting for 80–86% of the mean annual rainfall. Runoff, precipitation and evaporation are the key components of Hulun Lake’s long-term water balance (Fu et al., 2021). The highest water level of Hulun Lake was 545.2 m in 1991 AD, with an average area of 2,135 km^2^ and an average depth of 6.4 m (Figure 1C).

Hulun Lake hosts rich biodiversity, particularly in fish species, which are predominantly from the *Cypriniformes* to *Salmoniformes*. Although the number of fish species is limited, the fauna composition is relatively diverse (Mao et al., 2016). Phytoplankton in the lake include Chlorophyta, Cyanobacteria, Euglenophyta, Diatom, and Chrysophyta, with Chlorophyta accounting for 56.3% of the total phytoplankton. Benthic fauna primarily consists of *Macropelopia* sp., *Chironomus flaviplumus*, and *Limnodrilus hoffmeisteri* (Ma et al., 2021). Zooplankton include *copepods*, *cladocerans*, and *rotifers* (Zhao et al., 2022). The zonal vegetation around Hulun Lake is dominated by grassland, primarily composed of *Stipa grandis*, *Leymus Chinensis*, and *Stipa krylovii* communities. Lowland areas and riverbanks are characterized by Carex-forb grass meadows and *Phragmites australis* meadows (Xue et al., 2017).

### 2.2 Sampling and chronological analysis

In September 2022, a sediment core (HLH-22) with a length of 49 cm was collected using a gravity corer at a water depth of 3.6 m from Hulun Lake (49°03′59.21″N, 117°33′00.19″E). The core samples were stratified at 0.5 cm intervals at the site, and stored in clean ziplock bags at 4°C in the laboratory of the Nanjing Institute of Geography and Limnology, Chinese Academy of Sciences (NIGLAS). A total of 49 samples, at 0.5 cm intervals from 0 to 49 cm (selecting odd samples), were used for diatom, grain size, chronology, and other geochemical proxy analyses (e.g., total nitrogen, total phosphorus, total organic carbon).

For dating, an age model was developed using radiometric analyses of ^210^Pb and ^137^Cs. The activities of ^210^Pb and ^137^Cs in the sediment core were assessed with a high-purity germanium well detector at the NIGLAS laboratory. ^210^Pb in dried sediments was measured via gamma emission at 46.5 keV, while ^137^Cs was measured at 662 keV (Wan et al., 1987). The accuracy of the sediment core age derived from ^210^Pb dating models was validated using the fixed peak of ^137^Cs originating from intensive nuclear weapons testing around 1963 AD (Appleby, 2002). The chronology of the core was established based on the constant rate of supply (CRS) model (Appleby and Oldfield, 1978). The core reason for applying the CRS (Constant Rate of Supply) model in dating for Hulun Lake is the strong alignment between its dynamic sedimentary environment—characterized by significant variations in sedimentation rates and frequent disturbances—and the flexibility of the CRS model. This model effectively integrates the ^137^Cs marker layer to enhance the reliability of dating results, providing critical temporal benchmarks for revealing the evolution of the lake environment.

### 2.3 Subfossil diatom analysis

Approximately 0.30 g of dried sample was used for diatom analyses. All samples were treated with 10% HCl and 30% H_2_O_2_. The resulting slurries were washed repeatedly with distilled water, spread onto glass coverslips, and allowed to dry at room temperature before being mounted with high refractive index Naphrax^®^ (Battarbee, 2000). Diatom counts were conducted using an Olympus BX51 microscope with an oil immersion objective (magnification ×1,000). A minimum of 300 valves were counted per sample. Taxonomic identifications primarily followed Krammer and Lange-Bertalot (1986a, 1986b, 1991a, 1991b) and Houk (2010) with taxonomic nomenclature following Lange-Bertalot et al. (2017) and Ács et al. (2016).

### 2.4 Sediment geochemistry and grain size analysis

Samples from the HLH-22 core were analyzed for TN, TP, TOC, and C/N measurements. The total organic carbon (TOC) was determined by eliminating carbonates with 1 M HCl (Mei et al., 2024). Sediment TOC and TN were quantified using a CE-440 elemental analyzer (EAI Company Ltd), while TP concentrations were assessed using ICP-AES. C/N ratios were calculated on an atomic basis. Grain size analysis employed a laser size distribution apparatus with a measurement range of 0.02–2,000 μm (Malvern Mastersizer 3000, Malvern Panalytical Ltd., UK). Samples were pretreated with 10–20 mL of 30% H_2_O_2_ to remove organic matter, followed by treatment with 10 mL of 10% HCl to remove carbonates. After approximately 24 h, deionized water was added to rinse away acidic ions. The sample residue was then treated with 10 mL of 0.05 M (NaPO_3_)_6_ in an ultrasonic vibrator for 10 min to facilitate dispersion before conducting grain size analysis (Peng et al., 2005; Wang et al., 2020). All analyses were performed at the NIGLAS laboratory.

### 2.5 Statistical analyses

The diatom remains were included in the statistical analyses (at least one sample at >1%). Diatom abundances were square root transformed to stabilize variance (ter Braak and Šmilauer, 2002). The zonation of the diatom-based biostratigraphy was determined by cluster analysis using the constrained incremental sum of squares method (CONISS) within the Tilia 2.6.1 program, which was also used to create the diatom diagrams (Grimm et al., 1991).

Detrended Correspondence Analysis (DCA) was conducted to assess variation in diatom composition within the data. The DCA analysis revealed a maximum gradient length of 2.2, suggesting that a linear ordination model, such as Redundancy Analysis (RDA) would be statistically more suitable. RDA analyses were conducted with diatoms as response variables and geochemical data (e.g., TOC, TP, TN, grain size) as explanatory variables, utilizing Canoco 5.

To explore the dynamic changes in lake diatom communities and identify their driving factors, we employed Mantel tests to evaluate the impacts of temporal variation and paleoenvironmental traits on diatom community diversity. The environmental factors considered included TN, TP, TOC, C/N ratios, and grain size. Environmental distances were calculated using Euclidean distance with standardized variables, while community dissimilarity was assessed using weighted UniFrac distances. Following these calculations, Mantel tests were conducted based on correlations within the distance/dissimilarity matrices (Sunagawa et al., 2015). Statistical significance (*p* < 0.05) of the Mantel tests was determined using a permutation procedure repeated 999 times (Crabot et al., 2019). Correlations with *r* > 0.2 were considered valid associations, indicating the influence of environmental factors on diatom communities (Zhang C. et al., 2023; Zhang Z. et al., 2023).

## 3 Results

### 3.1 Chronology of sediment core

**Figure 2:**
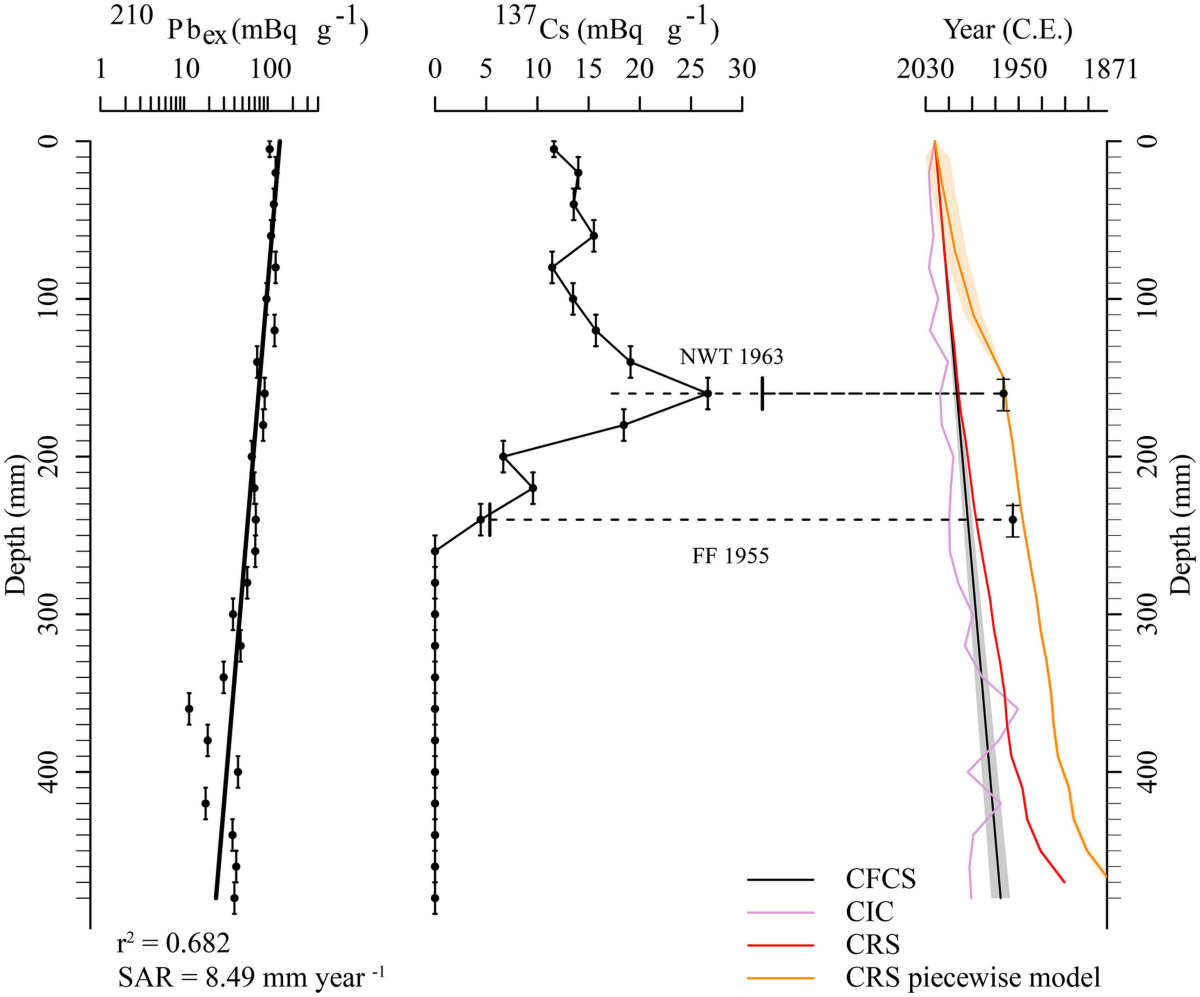
Age-depth model for the HLH-22 core from Hulun Lake, including variations in ^210^Pb_ex_ and ^137^Cs activities and sedimentary chronology.

The ^137^Cs curve in the HLH-22 core displays a typical northern hemisphere distribution pattern, with the peak value at 15.5 cm corresponding to the 1963-time scale (Figure 2). The ^210^Pb_ex_ activity in the sediment core shows a reasonable exponential decay proportional to depth. The ^210^Pb_ex_ dates were calculated using the constant rate of supply (CRS) dating model, validated with the ^137^Cs stratigraphic record, particularly the 1963-peak. Therefore, after ^137^Cs calibration, the ^210^Pb_ex_ CRS model of the HLH-22 core spanned a period of approximately 151 years (1871 to 2022 AD).

### 3.2 Subfossil diatom assemblages

**Figure 3:**
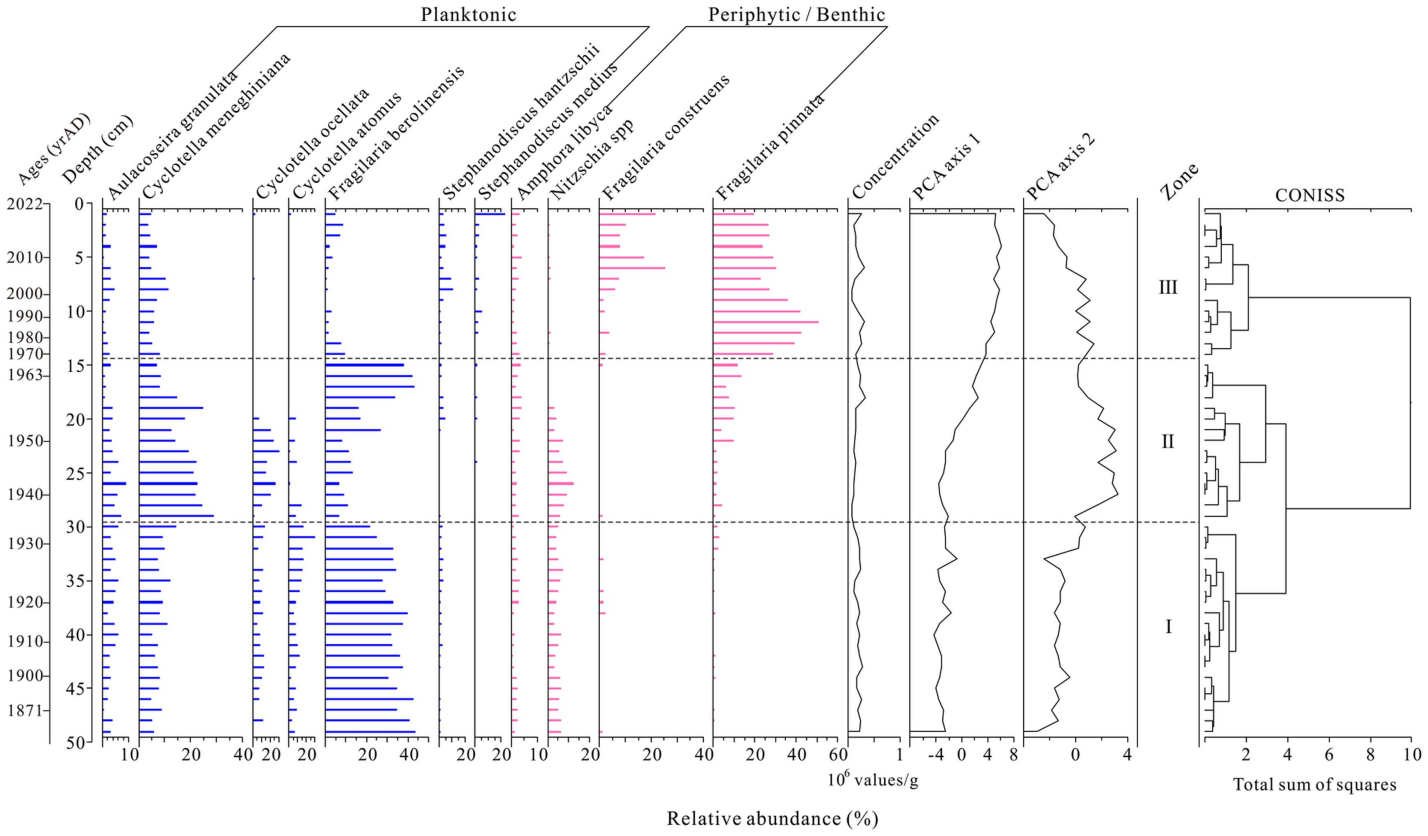
Diagram of diatom records from Hulun Lake over the past 151 years. Diatom taxa with a relative abundance >1% in at least one sample in the sediments are shown. Zones of major compositional change are indicated by horizontal lines.

Diatoms were abundant and well-preserved throughout the sediment record from Hulun Lake (Figure 3). A total of 114 diatom species, each with over 1% abundance, were identified, including 11 key taxa. The assemblages were dominated by planktonic species such as *Cyclotella meneghiniana*, *Fragilaria berolinensis*, and *Aulacoseira distans*, alongside small-sized benthic species like *Nitzschia* spp., *Fragilaria construens*, and *Fragilaria pinnata*. The CONISS cluster analysis identified three distinct diatom assemblage zones: Zone I (48.5–29.5 cm, ca. 1871 to 1935 AD) and Zone II (29.5–14.5 cm, ca. 1935 to 1970 AD) and Zone III (14.5–0 cm, ca. 1970 to 2022 AD).

#### 3.2.1 Zone I (ca. 1871 to 1935 AD)

This zone marks the initial changes in planktonic taxa, characterized by high abundances of *Cyclotella meneghiniana* and *Fragilaria berolinensis*. While planktonic taxa dominated in the early sedimental environment, the periphytic/benthic flora, particularly *Nitzschia* spp., quickly increased, comprising more than 10% of the assemblage.

#### 3.2.2 Zone II (ca. 1935 to 1970 AD)

During this period, *Cyclotella meneghiniana* continued to increase in abundance, while *Nitzschia* spp. remained prevalent. However, *Fragilaria berolinensis* showed a declining trend, with its mean relative abundance decreasing to 25%. In contrast, *Fragilaria pinnata* demonstrated an upward trend in abundance.

#### 3.2.3 Zone III (ca. 1970s to 2022 AD)

This zone is characterized by a significant increase in periphytic/benthic diatom species, particularly *Fragilaria pinnata*, at the expense of *Cyclotella meneghiniana*. Notably, this period also witnessed the abrupt decay of *Nitzschia* spp. and a marked increase in the relative abundance of *Fragilaria construens* and *Stephanodiscus medius*, alongside a decline in *Fragilaria berolinensis*.

### 3.3 Sediment geochemistry and grain size distribution

**Figure 4:**
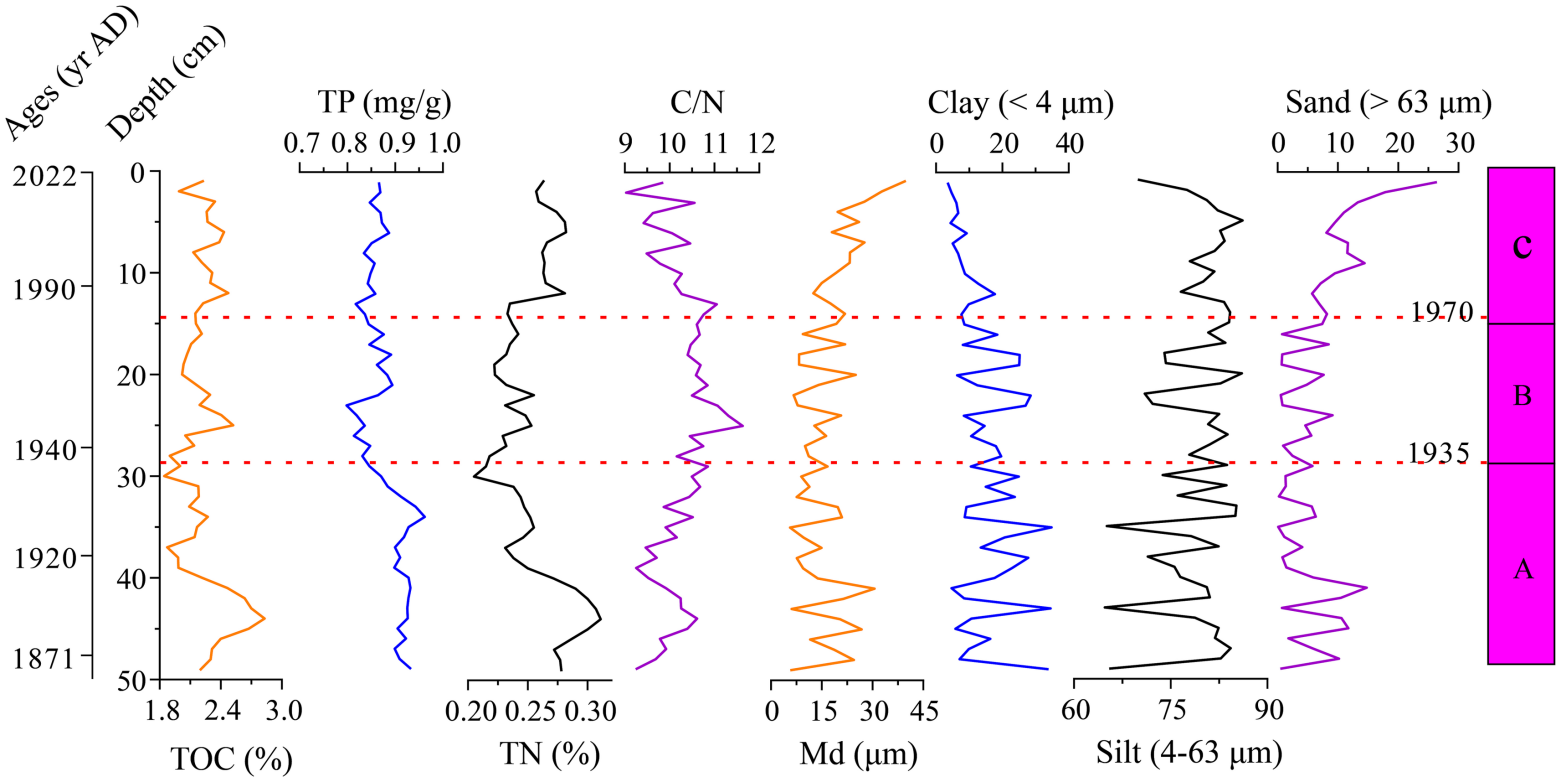
Time series of TOC, TP, TN, C/N over the past century and vertical distribution of grain size in the sediment core from the HLH-22.

The sediment proxy record of lake nutrients included TOC, TP, TN, and C/N ratios. TOC contents ranged from 1.84 to 2.83%, while TN varied from 0.21 to 0.31%, both exhibiting a similar historical trend with an increase after 1935 AD. TP levels remained relatively stable, ranging from 0.80 mg/g to 0.96 mg/g. The C/N ratio showed an overall increasing trend from 1871 to 1970 AD, followed by a noticeable decline (Figure 4). These variations in TOC, TP, TN, and C/N suggest significant changes in the trophic conditions of Hulun Lake over the past century.

The sediment core’s grain size distribution, including median diameter (Md), clay (<4 μm), silt (4–63 μm), and sand (>63 μm), is presented in Figure 4. In Hulun Lake, the distribution primarily consisted of silt (4–63 μm), contributing 64.77 to 86.15% of the total sediment, the clay (<4 μm) and sand (>63 μm) fractions accounted for 3.52 to 34.83% and 0.10 to 26.41%, respectively, the Md values ranged from 5.59 to 39.51 μm. Down-core variations of grain size parameters did not show distinct trends but were characterized by a significant period (ca. 1970 to 2022 AD), during which the content of the >63 μm size fraction and Md values increased significantly, while the <4 μm fractions decreased. From 1970 to 2022 AD, the >63 μm size fraction peaked at 26.41%, and the Md values reached a maximum of 39.51 μm.

### 3.4 Associations between environmental factors and diatom communities

RDA was conducted to explore the relationships between subfossil diatom taxa and environmental variables in core samples from Hulun Lake. The results indicate that both TP and sand were strongly correlated with variations in diatom abundance and composition. Notably, sand was positively correlated with the first RDA axis, which explained 51.47% of the variance and showed a negative correlation with most planktonic diatoms.

**Figure 5:**
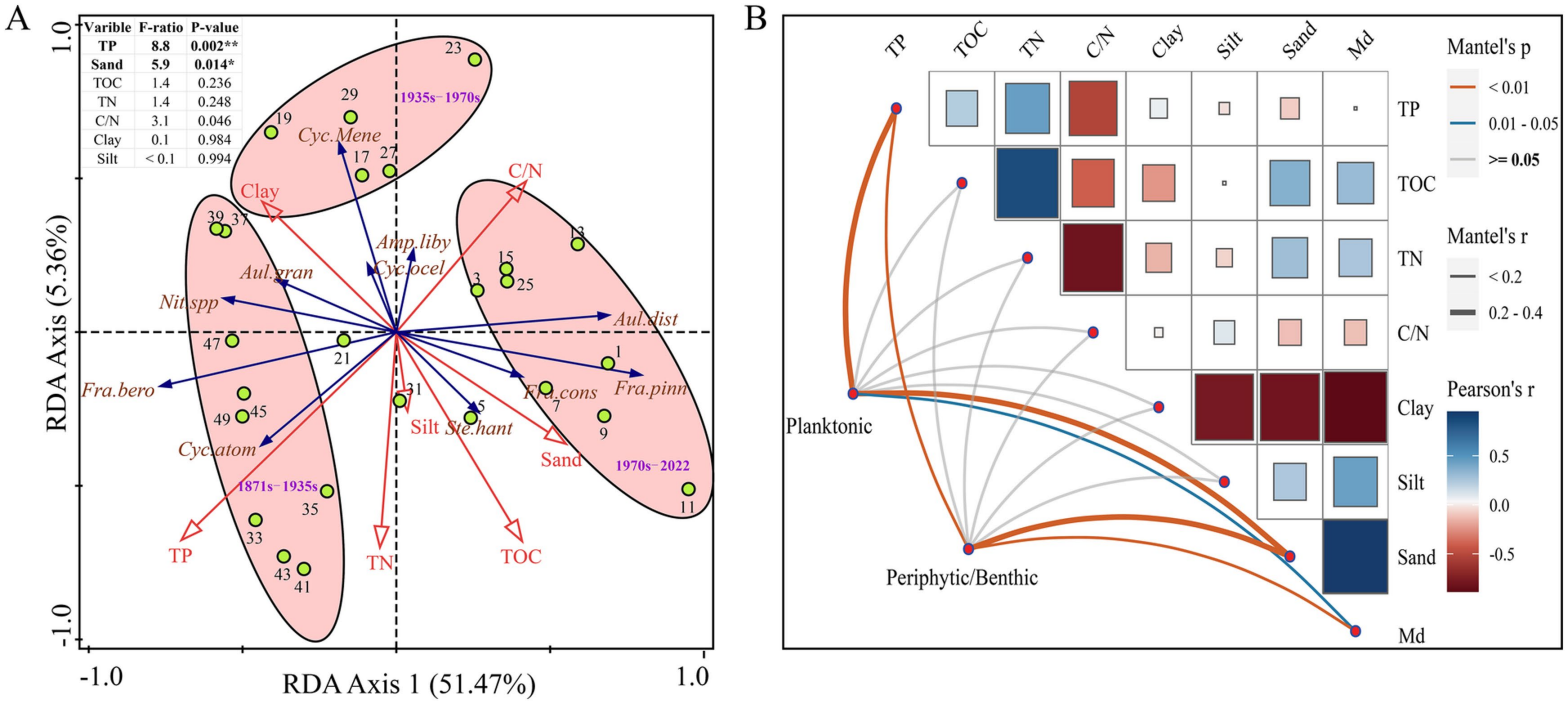
Putative functional profiles of planktonic and periphytic/benthic diatom communities and environmental parameters associated with diatom composition. **(A)** Redundancy analysis illustrating the relationships between the subfossil diatom taxa and environmental variables in core samples from Hulun Lake. **(B)** Pearson’s correlation coefficients of the main 8 environmental parameters (Euclidean distance), planktonic (Bray-Curtis distance), and Periphytic/Benthic (Bray-Curtis distance) of diatom communities using Mantel permutation test.

During the period from 1871 to 1935 AD, TP was positively associated with certain planktonic taxa (e.g., *Aulacoseira granulata*, *Nitzschia* spp., *Fragilaria berolinensis*, *Cyclotella atomus*) along the negative direction of Axis 1. The period from 1935 to 1970 AD was characterized by a limited presence of planktonic species. Axis 2 accounted for only 5.36% of the diatom variance and was negatively correlated with *Fragilaria construens* and sand. From 1970 to 2022 AD, the dominant species, *Fragilaria pinnata*, were found in the positive direction of Axis 1, consistent with the trend observed for sand (Figure 5A).

Further analysis using the Mantel test revealed associations between environmental factors and diatom communities at various successional stages of Hulun Lake (Figure 5B). The results demonstrated significant correlations between diatom communities in the sediments from Hulun Lake with TP and sand (*r* > 0.2, *p* < 0.01), followed by median diameter (Md) (*r* < 0.2, *p* < 0.01). Other environmental factors showed no significant correlation with diatom communities.

## 4 Discussion

### 4.1 Multiple indicators explanation of ecological environment changes in Hulun Lake

In many freshwater ecosystems, diatoms often dominate algal communities. Their communities are sensitive to environmental changes (Wang et al., 2020), could provide valueble evidence of long-term environmental shifts (Wohlfarth et al., 2008) and reflect the impacts of climate change and human activities on lake ecosystem (Liu et al., 2017; Smol et al., 2005). Over the past century, the ecological environment of Hulun Lake has undergone significant changes driven by both human activities and climate change (Sun et al., 2023; Wang et al., 2015). In this study, palaeolimnology records from core HLH-22 indicate distinct changes in diatom communities (Figure 3). Specifically, we identified three environmental stages (early, middle, and late) based on analysis of diatom community assemblages. The early stage (ca. 1871 to 1935 AD) is characterized by a generally high abundance of subfossil diatoms, predominantly *Fragilaria berolinensis*, a freshwater planktonic species commonly found in eutrophic lakes across both hemispheres (Round and Maidana, 2001). This suggests that Hulun Lake may have had higher nutrient levels during this period. The middle stage (ca. 1935 to 1970 AD) is marked by a continued high abundance of subfossil planktonic diatoms, with *Cyclotella meneghiniana* reaching its peak abundance (39.81%). This species is also considered an indicator of eutrophic conditions (Finlay et al., 2002), further suggesting that Hulun Lake remained in a eutrophic state during this period. The late stage (ca. 1970 to 2022 AD) is characterized by a shift toward an abundance of periphytic/benthic diatoms, with *Fragilaria pinnata* and *Fragilaria construens* becoming the most dominant species (Figure 3). These species are typically associated with shallow water environments. In contrast, the abundance of eutrophic species such as *Fragilaria berolinensis* and *Cyclotella meneghiniana* decreased, which indirectly indicates a reduction in both the water level and nutrient levels of Hulun Lake compared to earlier periods.

The contents of the nutrient elements such as TOC, TN, and TP in lake sediments are closely linked to lake productivity and are important indicators for studying the evolution of a lake’s nutrient status (Wu et al., 2007). Organic matter in lake sediments primarily originates from plant debris, including endogenous organic matter produced by aquatic organisms within the lake, as well as exogenous organic matter transported into the lake through rivers or surface runoff from the surrounding basin (Barnes and Barnes, 1978). Nitrogen in lake sediments is predominantly present in the form of organic nitrogen, which is mainly derived from lake organisms under natural conditions, although human activities can also contribute to nitrogen inputs in some lakes (Bechtel and Schubert, 2009). Phosphorus in lake sediments can be derived from both natural sources within the basin and from human inputs. Phosphorus from natural sources typically occurs in particulate form, while phosphorus from human sources primarily enters the lake in dissolved form, much of which is eventually accumulated in the sediments in a bioavailable state. This form of phosphorus has a direct impact on the lake’s nutrient status (Rydin, 2000). In the early stage (ca. 1871 to 1935 AD), we observed that TOC, TP, and TN were at high values (Figure 4) which is consistent with the presence of eutrophic diatom species, indicating that the nutrient levels in Hulun Lake were high during this period. During the middle stage (ca. 1935 to 1970 AD), the values of TN, TP, and TOC decreased relative to the early stage (Figure 4), though the lake remained in an overall eutrophic state. In the late stage (ca. 1970 to 2022 AD), TN and TP values exhibited a slow upward trend (Figure 4). Previous studies have identified two periods of increased TN and TP values in Hulun Lake: the late 1980 AD to early 1990 AD and around the 2010 AD (Han and Yang, 2002). These peaks further suggest that the nutrient levels in the lake remained relatively high during the late stage.

The coarseness or fineness of lake sediment particles can serve as an indicator of changes in lake area, reflecting shifts in the dry-wet climate conditions of the lake (Chen et al., 2003; Wang et al., 2010). Coarse-grained sediments are typically associated with dry climate periods, during which the lake area shrinks and the water becomes shallower, while fine-grained sediments are indicative of wet climate periods, when the lake expands and the water becomes deeper (Sun et al., 2018). During the early stage (ca. 1871 to 1935 AD), sediment grain size exhibited strong oscillations (Figure 4), suggesting that the sedimentary environment of Hulun Lake was highly unstable during this period. In the middle stage (ca. 1935 to 1970 AD), the sediment grain size continued to show oscillation characteristics, though these fluctuations were less pronounced compared to the earlier stage (Figure 4). In the late stage (ca. 1970 to 2022 AD), sediment grain size exhibited relatively stable characteristics, with the Md showing an upward trend. During this period, clay content decreased, sand content increased (Figure 4), and the water level of the lake dropped (Figure 1C), which is consistent with instrumental data.

### 4.2 Mechanism driving the long-term evolution of the ecological environment in Hulun Lake

Paleoecological records provide valuable insights into how ecosystems have responded to past environmental shifts, offering an alternative approach to assessing ecosystem sensitivity to environmental changes (Nolan et al., 2018). The mechanisms underlying the long-term ecological and environmental changes in Hulun Lake can be categorized into two main factors: natural processes and human activities. Based on the results of diatom community cluster analysis, we identified three distinct environmental stages: early, middle, and late.

During the early stage (ca. 1871 to 1935 AD), paleolimnological results indicated that the diatom community in Hulun Lake was primarily composed of eutrophic species, particularly *Fragilaria berolinensis*. Geochemical indicators, such as TOC and TN, were high around 1903 AD, and the C/N ratio was elevated, suggesting a significant input of terrestrial organic matter. These findings align with historical records, which describe Hulun Lake as a swamp during this period. The original lake basin consisted of several interconnected water bodies stretching in a northeast-southwest direction, surrounded by flat grasslands. Low-lying areas transitioned into reed ponds (Qin and Wang, 1994). The prevailing winds likely transported large quantities of tumbleweeds, plant detritus, and soils into the lake. The degradation of this material released nutrients, particularly phosphorus (which is consistent with the results of the RDA), thereby increasing the nutrient levels in the lake (Sun et al., 2018; Wang et al., 2020). Overall, this period likely reflects the influence of natural processes, with early human activities having minimal impact on the lake environment.

**Figure 6:**
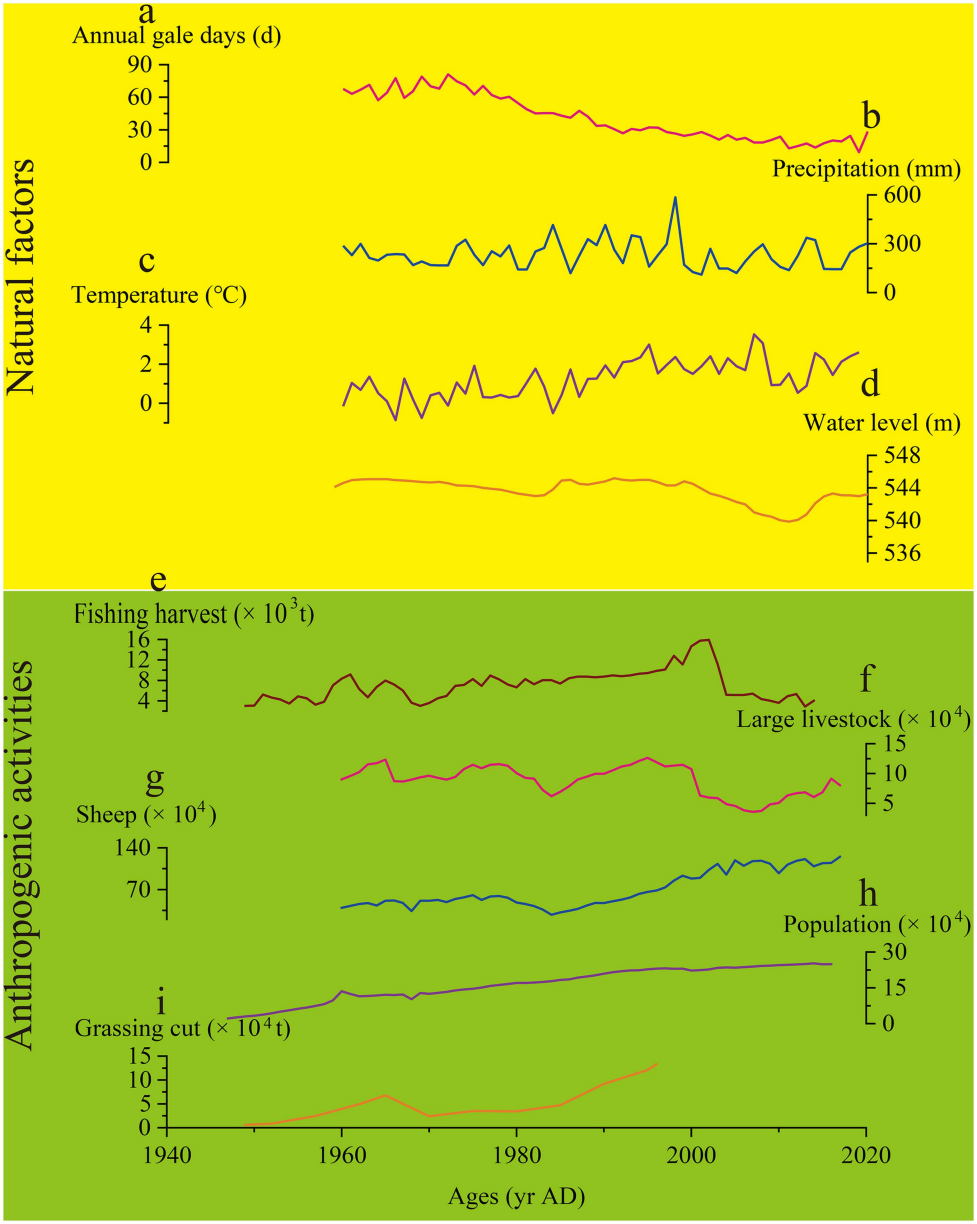
Time series of natural factors: **(a)** Annual gale days, **(b)** Precipitation, **(c)** Temperature, **(d)** Water level, and anthropogenic activities: **(e)** Fishing harvest, **(f)** Large Livestock, **(g)** Sheep, **(h)** Population, **(i)** Grassing cut in Hulun Lake over the past century. Data sourced from the National Nature Reserve Administration of Hulun Lake and the New Barhu Left Banner log and Research Report on the Ecological Environment of Chinese Lakes.

During the middle stage (ca. 1935–1970 AD), the eutrophic species *Cyclotella meneghiniana* significantly increased. At the same time, the values of TN, TP and TOC decreased compared to the early period, although the overall trend remained upward. This change may be linked to fluctuations in the water level of Hulun Lake. According to instrumental data, the water level remained high during this period (Figure 6). Some studies suggest that high water levels can lead to a decrease in nutrient concentrations, as the increased water volume dilutes the nutrients (Huang et al., 2023). Furthermore, the presence of aquatic plant communities along the northeast and southeast shores of the lake may have also played a role. High water levels likely promoted the growth of plants, such as reeds, which absorb nutrients, thus affecting the overall nutrient levels in the water (Chen et al., 2020). Except for these natural factors, the population of the Hulun Lake Basin grew significantly, from around 21,000 in 1947 AD to nearly 125,000 by 1970 AD (Figure 6). This population growth led to the direct discharge of untreated domestic sewage into the lake, contributing to water eutrophication (Yang et al., 2019). Additionally, the development of the Zhalainuoer mining area resulted in a significant nutrient influx into the lake (Yu et al., 2021). Although these led to increasing in nutrient levels, the overall trend still showed a drop in nutrients compared to the early stage. Therefore, the nutrient level of Hulun Lake was not high. During this period, the Md of the sediments showed an overall upward trend (Figure 4). Historical records indicate that the number of annual gale days in the Inner Mongolia Autonomous Region was relatively high, which caused soils, plant fragments, and hay from the exposed areas around the Hulun Lake Basin to be blown into the lake. Furthermore, as the population grew, the amount of grassing cut for grazing increased, and the number of livestock, particularly sheep, remained high (Figure 6). This, combined with an unsustainable grazing system, led to grassland degradation, which in turn affected the nutrient load in runoff from rainfall, increasing the input of exogenous nutrients into the lake. The ecological changes in Hulun Lake were further influenced by water use for industrial, agricultural, and domestic purposes, as well as the construction of water conservancy projects, which had the greatest and most direct impact on the lake’s hydrology. In 1963 AD, a dam was built in the Zhalainuoer mining area, blocking the outflow channel of the lake. This caused the water level to reach its highest historical point during the 1960 to 1970 AD, which was influenced by both human activities and climate change (Cai et al., 2016). In summary, the ecological changes in Hulun Lake during this stage were driven by a combination of natural factors and human activities. The sedimentary environment was not very stable, reflecting the complex interplay of these influences.

The late stage (ca. 1970 to 2022 AD) is characterized by a substantial shift in the diatom community composition in Hulun Lake sediments, transitioning from primarily planktonic to periphytic/benthic taxa. Our results are consistent with instrumental data, particularly after 2000 AD, which show a general downward trend in the water level of Hulun Lake. During this period, TN and TP exhibited a slow upward trend (Figure 4), primarily due to ongoing eutrophication in the lake. The population around Hulun Lake has remained relatively sparse, with areas such as New Barhu Left Banner, New Barhu Right Banner, and Manzhouli showing only a modest increase of over 20,000 people since the 2000 AD. Grazing activities, particularly grassing cut, have also increased, with sheep numbers remaining high, while the number of large livestock has remained low (Figure 4). This suggests that human activities have had a limited impact on the eutrophication of the lake, with the primary driver of nutrient levels being changes in the water volume, influenced by climate change. Fishing harvest data further support this view. The fishing harvest has shown a sharp decline since 2000 AD, likely due to eutrophication-induced changes in the lake’s biological food base. Increased nutrient levels promote the rapid growth of phytoplankton, such as algae, which can alter the aquatic food web and negatively impact fish populations (Mao et al., 2016). Previous studies have indicated that the late 1980s to early 1990s marked a peak in TN and TP concentrations in Hulun Lake (Han and Yang, 2002). At the same time, climate warming and drought conditions further accelerated vegetation degradation and soil erosion, leading to an influx of nutrients into the lake and a deterioration of the ecological environment (Yan et al., 2005). Since 1970 AD, significant changes in sediment particle size have been observed, especially in the sand content, which has shown a clear upward trend. Some studies suggest that heavy rainfall and increased water flow from upstream have raised the carrying capacity of the flowing water, leading to the transport of coarser particles into the lake (Xu et al., 2015). Additionally, the period’s warming and drying climate caused a significant drop in water levels, lake area shrinkage, and increased wind-driven erosion, all of which contributed to the coarsening of the sediment particles in Hulun Lake. Changes in sediment particle coarseness and fineness are often used to reflect the contraction and expansion of the lake surface. In this paper, with high sand content indicating a shallow water level and an unstable sedimentary environment with strong hydrodynamic conditions (Yu et al., 2016). Furthermore, some diatom species are especially sensitive to water level changes, supporting the idea that hydrological variability is a key factor driving diatom succession (Benoiston et al., 2017). These findings are further corroborated by results from the Mantel test. In summary, after 1970 AD, compared with human activities, natural factors have played a dominant role in the impact on the ecological environment of Hulun Lake.

### 4.3 Comparison with other semi-arid lakes

The ecological and environmental changes in Hulun Lake are not isolated but are part of a broader pattern observed in semi-arid lakes. For instance, studies on other semi-arid lakes such as Qinghai Lake and Daihai Lake have also shown significant responses to climate change and human activities (Chen et al., 2021; Sun et al., 2006). Qinghai Lake, located in the Qinghai-Tibet Plateau, has experienced significant changes in its ecological environment due to climate warming and human activities, leading to changes in lake water levels and nutrient loads (Hou et al., 2023). Similarly, Daihai Lake in the Inner Mongolia region has also shown significant changes in its ecological environment, with the lake area shrinking and water levels dropping due to increased human activities and climate change (Du et al., 2022). These studies highlight the common challenges faced by semi-arid lakes, such as the impact of climate change on water resources and the need for sustainable management practices. The findings from Hulun Lake are consistent with these broader trends, emphasizing the importance of understanding the complex interactions between natural processes and human activities in shaping the ecological environment of semi-arid lakes.

## 5 Conclusion

This study provides a comprehensive analysis of the ecological evolution of Hulun Lake over the past century, based on paleolimnological indicators, geochemical data, and diatom community composition. Our findings reveal a clear temporal shift in diatom assemblages, from predominantly planktonic species to periphytic/benthic species, which aligns closely with changes in nutrient levels and hydrological conditions. These results underscore the sensitivity of Hulun Lake’s ecosystem to environmental variations driven by both natural and anthropogenic factors. Our analysis identifies TP and sand content as key drivers of diatom community structure, reflecting the roles of nutrient influx and water-level fluctuations. Before 1935 AD, natural factors variability was the primary influence on Hulun Lake’s ecology, favoring eutrophic conditions. The period from 1935 to 1970 AD marked a gradual increase in nutrients due to human intervention but its ecological environment was still dominated by natural hydrological shifts. However, since 1970 AD, as human activities decrease and alterations in the lake’s outflow system, which have increasingly impacted Hulun Lake’s ecological dynamics, driving shifts in both nutrient levels and diatom community composition. Furthermore, the warming and drying trend of rising temperature, reduced precipitation has led to a significant drop in the lake water level and a shrinking water area. This shows that the eutrophication level of Hulun Lake is mainly controlled by the lake water volume caused by climate change. In conclusion, this study highlights the complex interplay between climate change and human activities in shaping the ecological environment of Hulun Lake. As global climate variability and regional human pressures continue to intensify, understanding the mechanisms driving lake ecosystem evolution is crucial for informing conservation and management strategies aimed at protecting these vulnerable freshwater resources.

## Data Availability

The raw data supporting the conclusions of this article will be made available by the authors, without undue reservation.
